# Measuring Evolutionary Isolation for Conservation

**DOI:** 10.1371/journal.pone.0113490

**Published:** 2014-12-10

**Authors:** David W. Redding, Florent Mazel, Arne Ø. Mooers

**Affiliations:** 1 Department of Biology, Simon Fraser University, Burnaby, BC, Canada; 2 Centre of Biodiversity, Ecology and Conservation, Department of Genetics, Evolution and Environment, University College London, London, United Kingdom; 3 Laboratoire d’Ecologie Alpine, Grenoble, France; Texas A&M University, United States of America

## Abstract

Conservation planning needs to account for limited resources when choosing those species on which to focus attention and resources. Currently, funding is biased to small sections of the tree of life, such as raptors and carnivores. One new approach for increasing the diversity of species under consideration considers how many close relatives a species has in its evolutionary tree. At least eleven different ways to measure this characteristic on phylogenies for the purposes of setting species-specific priorities for conservation have been proposed. We find that there is much redundancy within the current set, with three pairs of metrics being essentially identical. Non-redundant metrics represent different trade-offs between the unique evolutionary history represented by a species verses its average distance to all other species. Depending on which metric is used, species priority lists can differ as much as 85% for the top 100 species. We call for some consensus on the theory behind these metrics and suggest that all future developments are compared to the current published set, and offer scripts to aid such comparisons.

## Introduction

Conservation science has to make important decisions about which threatened species to conserve given the shortfalls in funding for many countries [1]. Although conservation action is often dictated by attributes such as body size, taxonomic grouping and social preference [2], objective measures are possible, e.g. threat status [3]. Here we consider one objective class of metric for guiding conservation attention that is gaining some traction: the isolation of a species on a phylogenetic tree [4], [5].

Some active conservation programmes have considered how to use evolutionary isolation to prioritise species. The US ‘Endangered Species Act’ of [6] gives priority during funding allocations to species that are monotypic within their genus over non-monotypic species, and the priority of both these groups over subspecies. Here, taxonomic information is used a substitute for detailed phylogenetic hypotheses. With more detailed hypotheses come many possible metrics that attempt to capture evolutionary isolation. These metrics use or combine information from the distance separating a species from the rest of the tree, the tree’s topology, and the distances between pairs of species on a phylogeny [7].

One particular phylogenetic evolutionary isolation metric (‘Fair Proportion’ [8]; termed ‘Evolutionary Distinctiveness’ in [9]) was adopted by the EDGE (Evolutionary Distinct and Globally Endangered) programme in 2007 [9] as part of a two-component ranking score (alongside a value for threat of extinction). At the time there was limited discussion of why this metric was used over others that were available and the effect of using a different isolation metric on prioritisation ranks in this context is unknown.

To aid conservation practitioners and researchers to choose which evolutionary isolation metrics to use in the future, we undertook the following simple study: we collated all the methodological options currently available and used a large simulated data set to compare scores and eliminate redundancy. We then investigated one specific cause of differences among evolutionary isolation scores: In parallel literature on functional diversity in ecology, a distinction has been made between the ‘uniqueness’ dimension of a species (i.e. its distance to the nearest relative in the functional space) and its ‘originality’ (i.e. its average distance to all other species in this space) [10]. In a phylogenetic context we can also use this distinction, with the ‘uniqueness’ being measured simply as the length of the pendant edge that separates a species from the rest of the tree, and ‘originality’ being characterised as the mean patristic distance to other all other species in target clade. Therefore, we ask how each of the known metrics projects on the two axes of “uniqueness” and “originality”.

Finally and against this background, we ask about the impact of differences in metrics on on-the-ground decision making by comparing the top 100 highest scoring mammal and amphibian species using EDGE-type scores created with all the different non-redundant isolation metrics. It turns out that the lists would be very different with different metrics, meaning we need a clear view of what we want to capture when measuring evolutionary isolation.

## Methods

We collated all the methods for measuring evolutionary isolation for creating species-specific conservation priorities from the literature (named and defined in Table 1) [11]–[17]. To aid simulation work, all metrics were first cast in a common analytical framework (Table 1). We then created sets of simulated phylogenies (physim function in R package *phytools*) [18] to test how the scores computed using the different metrics differed on a variety of tree shapes. We created and report results for four principal sets of 1000 100-tip homogeneous birth-death trees, all with birth rate *λ* = 0.5, and with death rates µ = 0, 0.125, 0.25 and 0.4. For each tree in a simulation set, the eleven isolation metrics were calculated and standardised by dividing by the respective mean score. We included several metrics known *a priori* to be similar for completeness sake. For each group of standardised scores we then used an agglomerative clustering approach to create a Euclidean distance matrix among the 11 metrics, and then visualized this matrix using agglomerative hierarchical clustering (*agnes* function in R package *cluster*) [19]. This approach allows us to visualise, on each of the five tree distributions, those measures that are most similar versus those that measure different aspects of evolutionary isolation. We also compared metrics overall by creating a single 50% majority rule consensus tree from each of the 1000 agglomerative hierarchical trees representing distances among metrics for each simulation set (*consensus* function in R package *ape*) [20].

**Table 1:** Evolutionary isolation scores used in this study.

Scores	Description	Definition
Pendant Edge(PE, [11])	The minimum phylogeneticdistance between species*i* of edges non-incidentwith the *i^th^* species.	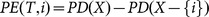
Shapley Value(SV [12])	Shapely Value for species *i* isthe expected decrease in *PD*species *i* causes when removedfrom an equiprobable set *X’* ofextant species of size 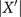 ,where *X’* is a subset of *X*.	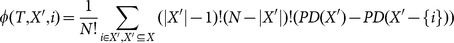
Fair Proportion(FP[8]) EvolutionaryDistinctiveness(ED [9])	FP for species i is the sum ofedge lengths along the pathfrom i to the root, each edgedivided by the number ofspecies ultimately subtending it	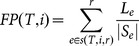
Equal Splits(ES [13])	Similar to ED, but each edgelength is divided by thenumber of the sister lineagese.g. 2 for a strictly bifurcating tree. For a bifurcating tree, edges with length *L_e_* that are *n*nodes away from a leadingtarget species [tip] *i* areproliferated by 0.5*^n^*.ES forspecies *i* is the sum of scoresattributed to each *L_e_* betweenspecies *i* and the root.	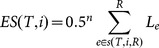
Vane-Wright(VW [4])	VW for species *i* is thereciprocal of the sum ofnodes *d* on the path betweenspecies *i* and the root.	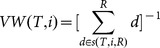
May-Vane-Wright (MVW, [14])	Similar to VW, MVW forspecies *i* is the reciprocal ofthe sum modification of VWof the number of daughterlineages *l[d]* that originatefrom all metric nodes *d* on thepath from species *i* and theroot. For a bifurcating node,the number of daughterlineages *l[d]* are 2.	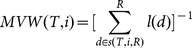
Nixon & Wheelerunweighted index(NWU [15])	A node *d* that descendsrelatively more species in asubclade is assigned a valueof 1 when compared to itssister node, otherwise 0[binary variable *b[d]* isassigned at all nodes *d*].NWU for species *i* is thereciprocal of the sum of theassigned values at allnodes between species*i* and the root.	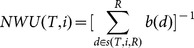
Nixon & Wheelerweighted index(NWW [15])	NWW is a modification ofNWU, where nodes *d* areassigned a count of theirtotal descendent species[*t[d]*]. NWW for species *i*is the reciprocal of the sumof the attributed values atall nodes between species*i* and the root.	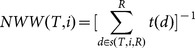
Average Pairwise Distance (APD [16])	APD for species *i* is theaverage pairwisephylogenetic distancebetween species *i* and allother species on a tree	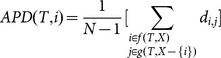
QE based index(QE [7])	QE is measured as thefrequency distributionwhich maximizes *Q*:	Let λi be the isolation of species i and QE = [QE1, QE2,…,QEN] the distribution of isolations of all species.
	*Q*[QE] = max [*Q*[p]], *Q*being the Rao quadraticentropy. Note that QEbased index	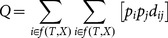
	might set scores to zerofor some species, if thetree is not ultrametric.	p_i_ and p_j_ are species relative weights and d_ij_ is the phylogenetic pairwise distance between species *i* and *j*.
		QE can be calculated as: 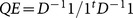
		with D being the matrix of phylogenetic pairwise distances d_ij_.
Character Rarity(CHR [17])	CHR for a species *i is* theproduct of the total novelcharacters 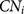 ,theirinheritance rate 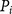 from aparticular node *k* to species*i*, and their expected rarityamong the sister speciesat that node.	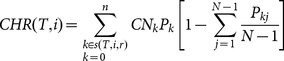

Let *T* be a rooted ultrametric phylogenetic tree with a root *R* and *X* as a set of species [leaves]. Further, let *N* be the size of *X*, and *n* be the total number of nodes in *T*.

We then examined the relative contribution of each metric to two dimensions of a species’ position in its tree: its ‘uniqueness’ (its pendent edge length) and its ‘originality’, (measured in this case as the mean patristic distance to all other species).

We did this projection in the following way: For each simulated tree (from the 100 tip tree set with *λ* = 0.5 and µ = 0.25), we first created two sets of 11 correlations scores: one from comparing the 11 metrics scores for each tip to each tip’s pendant edge value (spearman rank correlation, *cor* function in R package stats) [21] and a second from comparing the 11 metrics scores for each tip to each tip’s mean pairwise distance value (spearman rank correlation, *cor* function in R package stats) [21]. To prevent double representation of the same evolutionary isolation method, however, correlations for three metrics shown to be identical to others (see below) were subsequently dropped from each data frame [FP, VW & CHR], resulting in two sets of eight correlations per tree.

We then tested the influence the two evolutionary isolation concepts (‘uniqueness’ and ‘originality’) had on each of the 8 metrics by comparing these correlations. If the metrics differentially capture these two facets of isolation, the relationship between the two correlations across the 8 metrics should be strongly negative across all trees. This was tested using simple linear models (*lm* function from R package *stats*) [21] between the two sets of eight correlation scores.

Finally, we used the first two major groups assessed by the EDGE project, the mammals [22] and amphibians [23], to compare the top 100 species EDGE lists created using each of the eleven evolutionary isolation scores. To these scores, each metric of evolutionary isolation was first standardised and then substituted for the ED component in the EDGE formula [9], with the threat score taken from the IUCN red list [24] and name-matched using available taxonomies [25], [26]. All species were ranked on the eleven metrics and lists of the top 100 highest scoring species were compared for rank similarity (*cor* function R package *stats*) [21], and for the number of species they had in common with the original published EDGE list for that group.

## Results

### Relationship among metrics

Some pairs of metrics were always very closely related – the pairings of VW/MVW, ED/SV and APD/CHR (see Table 1 for metric acronym definitions) all had mean distances of 0, 0.04 and 0.12 (respectively) on the clustering trees where there is an expected pairwise distance of 1. Conversely, the most different metrics were APD and any members of the group {ES, ED/SV, PE}, which had a mean distance of approximately 1.56 units across the sets of clustering trees.

**Figure 1:**
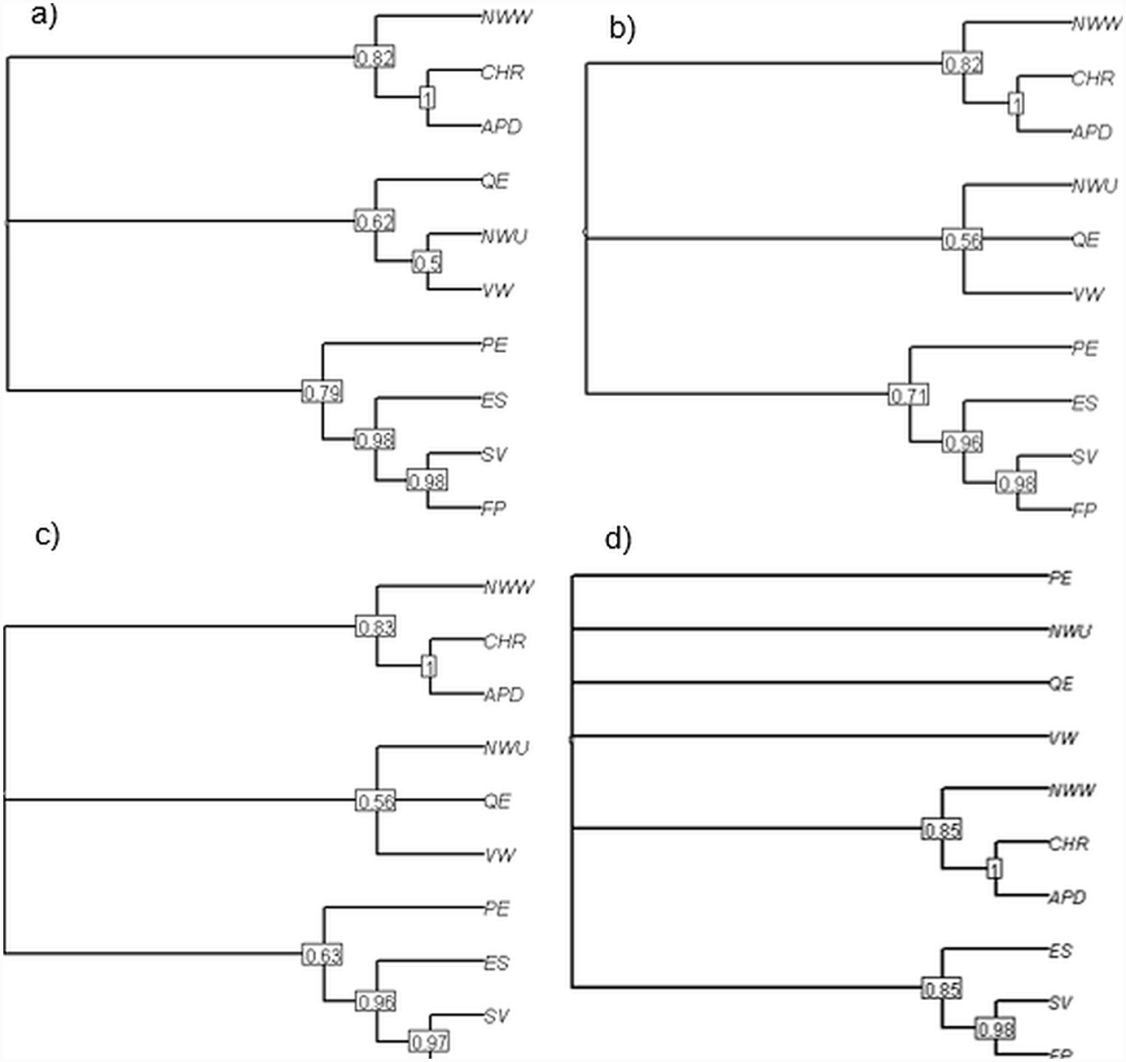
50% majority rule consensus trees show most common groupings of evolutionary isolation metrics based on distance between scores on when computed on 1000 100-tip random birth-death trees with a birth rate of 0.5 and death rates of a) 0, b) 0.125, c) 0.25 and d) 0.4. Scores in boxes represent the proportion of trees showing that grouping. Metric acronyms are described in Table 1.

**Figure 2:**
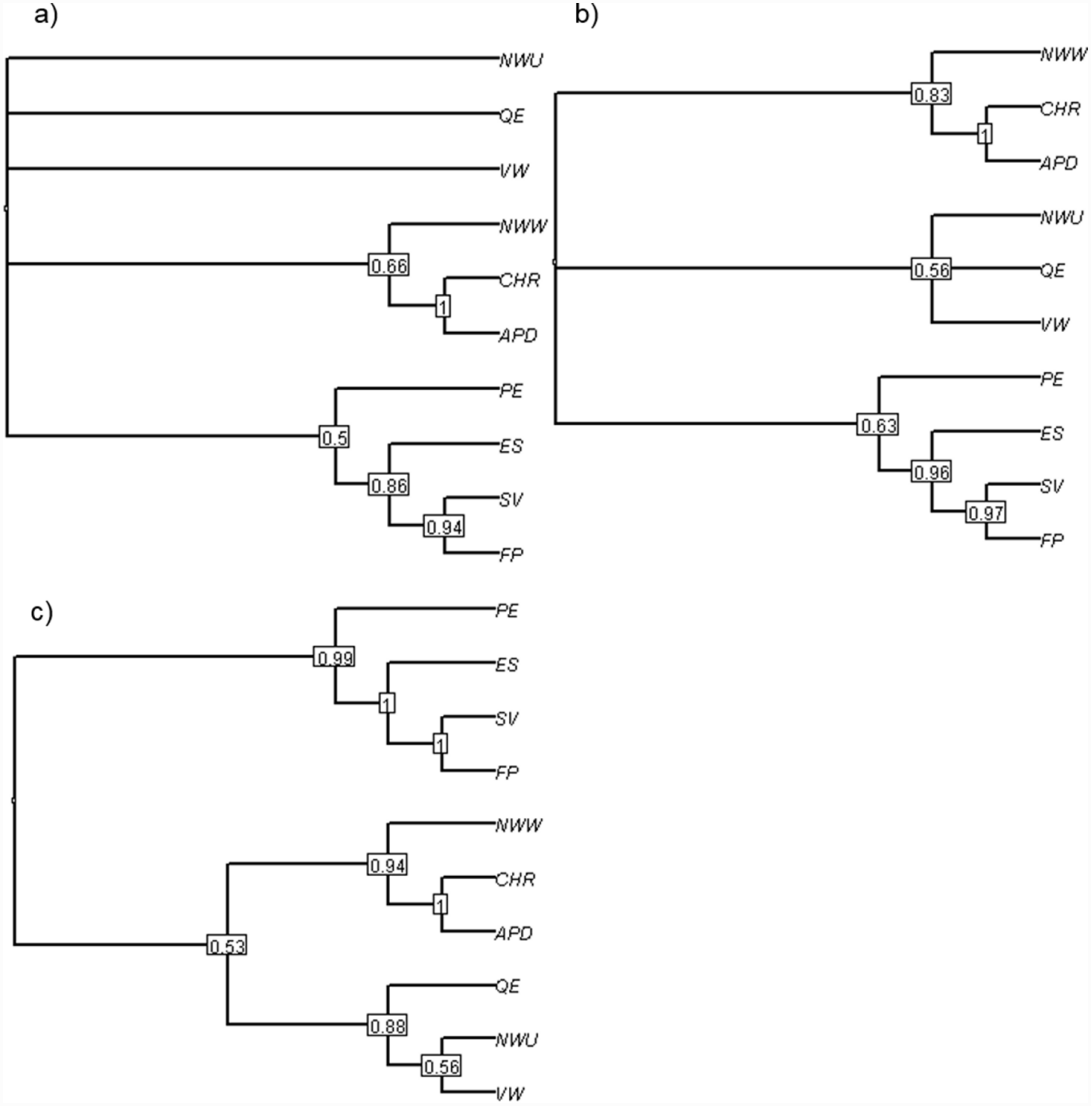
50% majority rule consensus trees show most common groupings of evolutionary isolation metrics based on distance between scores on when computed on 1000 random birth-death trees with a birth rate of 0.5 and death rates of 0.25 with a) 50 tips, b) 250 tips, c) 500 tips. Metric acronyms are described in Table 1.

The clusters emerging from each of the five simulated datasets supported, or at least did not contradict, the following principal groups: {ES, ED, SV, PE}, {NWW, APD, CHR} and {QE, VW, MVW, NWU} (for consensus trees see Fig. 1). In general, the latter group most often represented a ‘middle ground’ between the two other groups. Metric groupings calculated on simulated trees where birth and death rates were closer to parity (and so with shorter pendant edges) showed more variability, as indicated by the generally lower support values on the consensus clustering trees, with less than 50% support for the {QE, VW, MVW, NWU} grouping (Fig. 1d). The size of the trees had a similar effect; though the clustering trees were otherwise similar, the grouping of {QE, VW, MVW, NWU} was not recovered on more than 50% of trees for n = 50 (Fig. 2).

**Figure 3:**
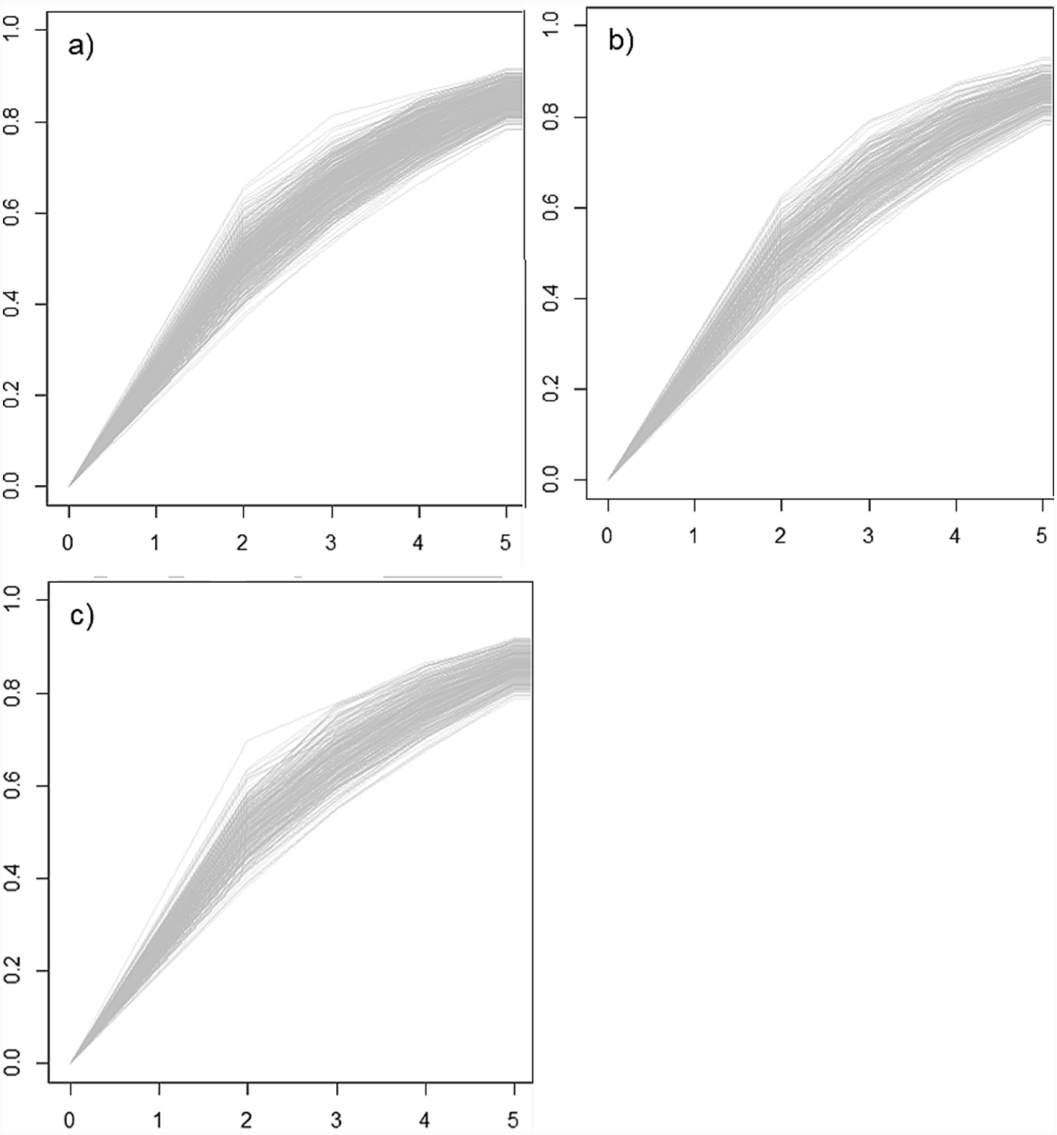
Total combined variation from all 11 different evolutionary isolation metrics that is captured when selecting the top 1 to 5 most different metrics, on sets of 1000 simulated random birth-death trees with a birth rate of 0.5 and death rates of a) 0, b) 0.125, c) 0.25 and d) 0.4.

Sets of the three most distantly related evolutionary isolation metrics (defined as a metric chosen randomly from each of the major groups on the majority-rule consensus clustering trees) captured on average 67% of the total variation in evolutionary isolation scores. In comparison, groups of the most dissimilar two, four and five metrics captured, approximately 51%, 78% and 86% of the total variation in scores respectively (Fig. 3). In the majority of cases the remaining six metrics in total only captured, on average, 14% of the remaining variation in scores, with the last three metrics adding minimal new information (<1% on average).

### Relationship to pendant edge and mean pairwise distance

**Figure 4:**
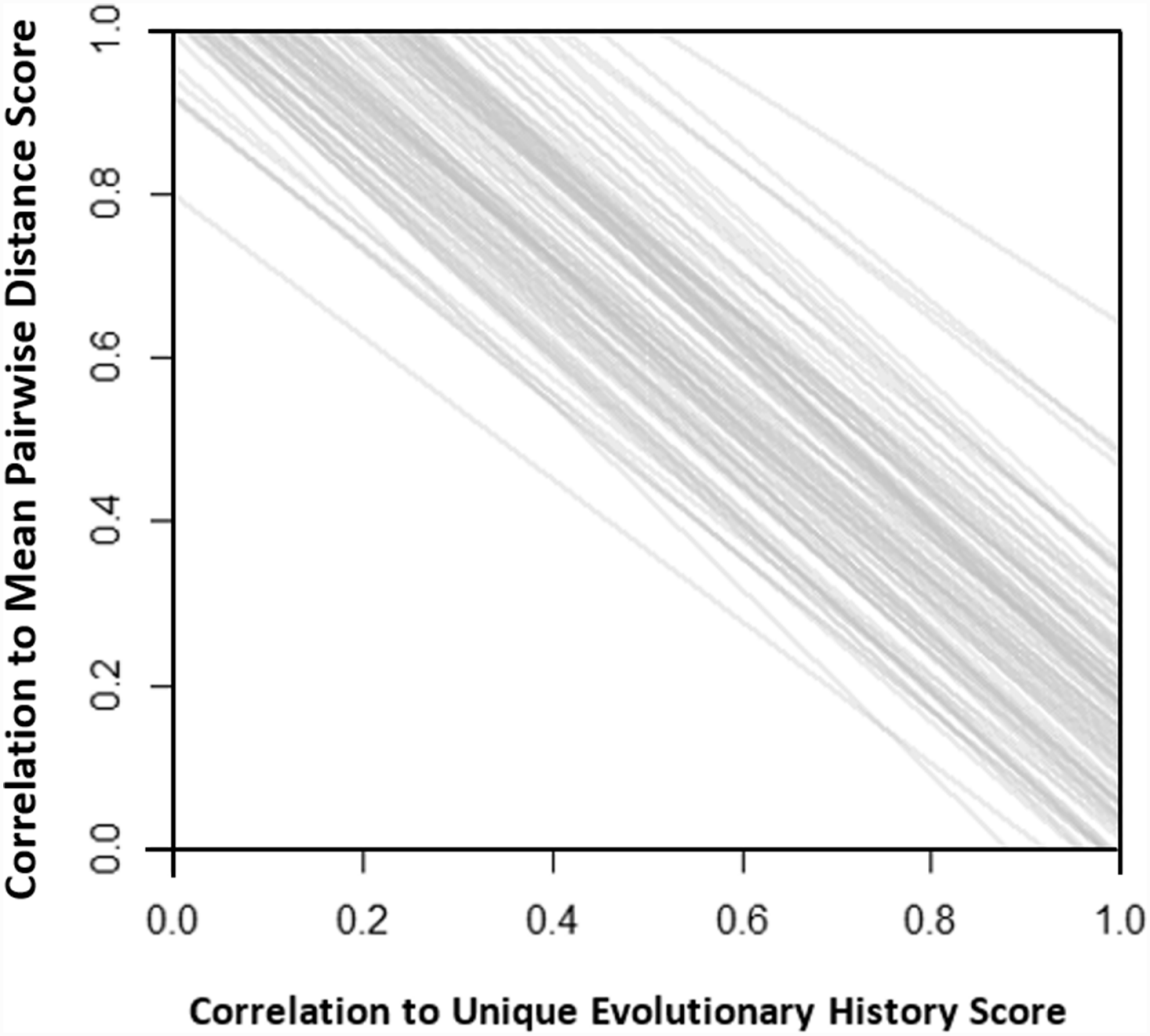
Fitted lines from linear models describing relationship between pairs of correlation scores (8 pairs of data points per line) across 1000 randomly simulated 100-tip trees (Birth-death 100 tips *λ* = 0.5 and µ = 0.25). The average adjusted R^2^ across the 1000 trees = 0.9, s.d.  = 0.07. Correlations were calculated between each tip’s evolutionary isolation scores (8 per tree) and two sub-components of evolutionary isolation: The amount of unique evolutionary history a species possess and the mean pairwise distance to all other species.

If an evolutionary isolation metric was strongly correlated to one axis of evolutionary isolation (unique evolutionary history or mean pairwise distance) it was generally weakly aligned to the other. This is illustrated by average slope of the linear models (Fig. 4) of the correlation scores of −1.06 (sd 0.04; n = 8; 1000 replications; average *adjusted* R^2^ 0.9 (sd = 0.07)).

### EDGE lists

The set of 11 evolutionary isolation metrics created dissimilar top 100 EDGE lists with several rank correlations for paired metrics being close to 0.5, and only ∼50% species shared on the top 100 list for a pair. Reassuringly, the metrics demonstrated to be most similar in the clustering analysis on simulated trees to the metric ‘ED’ produced very similar or identical top 100 EDGE-type lists on the real trees (Table 2).

**Table 2:** The relationships between EDGE lists using eleven different metrics of evolutionary isolation to create EDGE-type lists of the world’s Mammals and Amphibians.

	Mammals (n = 4920)			Amphibians (n = 5713)		
Metric	SharedSpecies	RankSimilarity	UniqueScores	SharedSpecies	RankSimilarity	UniqueScores
ED/FP	-	-	2326	-	-	1271
SV	100	1	2327	97	1	1271
ES	79	0.564	2291	92	0.907	1270
PE	78	0.667	544	90	0.894	1140
MVW	55	0.401	124	79	0.757	139
VW	55	0.401	124	79	0.757	139
QE	49	0.51	2055	59	0.435	1367
APD	47	0.447	2310	57	0.528	1271
CHR	47	0.44	2339	57	0.594	1271
NWW	44	0.146	419	57	0.515	368
NWU	53	0.237	119	41	0.494	148
Genus	50	0.42	187	15	0.667	278

‘Shared Species’ and ‘Rank Similarity’ (Spearmans ρ) are in comparison to the top 100 species in the original ED/FP list [9], [23]. ‘Unique Scores’ are the number of species with different scores when using that metric across all species.

## Discussion

We suggest that the metrics are all located somewhere on an axis that at one end is dominated by the distance of a species to all others within the tree {APD} and at the other end dominated by amount of unique evolutionary history {ED, SV} a species possesses. A third cluster of metrics {NWU, MAY & QE} appears to measuring a combination of these aspects of evolutionary isolation. Uniqueness and isolation [or average pairwise distance] have recently been well defined in a functional context [9], and APD and PE are the logical extremes of this axis. The strong relatedness between APD and NWW (the latter metric effectively measures the redundancy of species’ internal branches, on a path from root-to-tip) may help aid intuition of what APD actually captures [27]. However, a full analytical investigation of the metrics in this framework would be welcome.

In this framework, ED & SV weight the most important phylogenetic information as that near the tip, while MAY and NWU do not specifically weight tip information highly but only consider information from along a path from tip to root. Finally APD and NWW consider phylogenetic information from across the entire tree and have little emphasis on information from nearer the tips. We consider our results in more detail below.

### Redundancy

Many of the current metrics of evolutionary isolation appear to capture similar information: to capture most of the variation in evolutionary isolation as measured here, only a sub-set of the total number of metrics are required. The three very similar pairs of the metrics we demonstrate here are similar for different reasons: The close relationship between the Vane-Wright and May-Vane-Wright scores is unsurprising as the May-Vane-Wright function is simply a variation designed to cope with polytomies (Table 1). The close relationship between Average Pairwise Distance (APD) and Character Rarity (CHR) likewise has already been demonstrated [17]; we note that CHR will vary depending on supplied input values and only the standard ones were used here. The relationship between CHR and the others methods and the degree to which reasonable estimates for mutation and substitution rates for empirical species groups will affect its relationship to other metrics is an avenue for future study.

The redundancy between the Evolutionary Distinctness (ED) and Shapley value (SV) metrics is superficially surprising because the ED metric was created as an ad-hoc algorithm to allocate a phylogeny uniquely to its tips such that the most isolated species were allocated a greater proportion of the tree [8]. The Shapley value alternatively was derived from game theory where species are assessed on their potential to add branch length to future possible versions of evolution trees [12]. However, it is now known that the Shapley value converges on ED as trees become large [28]. This presents the opportunity to use the Shapley algorithm to compute a close proxy for ED. The main benefit here is that the Shapley value can be calculated on un-rooted trees and networks, providing greater flexibility [29].

As a set, many metrics of evolutionary isolation are redundant, or close to redundant, so it would be beneficial to focus any future work on a more concise set and ensure that any proposed novel metrics are examined against this set for existing redundancy. For example, we note that a recent metric presented as the “local” contribution of a tip to the matrix-wide “beta diversity” of a collective [30] can be cast as an isolation metric and can be shown to be proportional to the sum of all pairwise distances divided by the total sum of pairwise distances, i.e. is redundant with APD (for more details contact authors).

### Tree size and changes to evolutionary isolation scores

**Figure 5:**
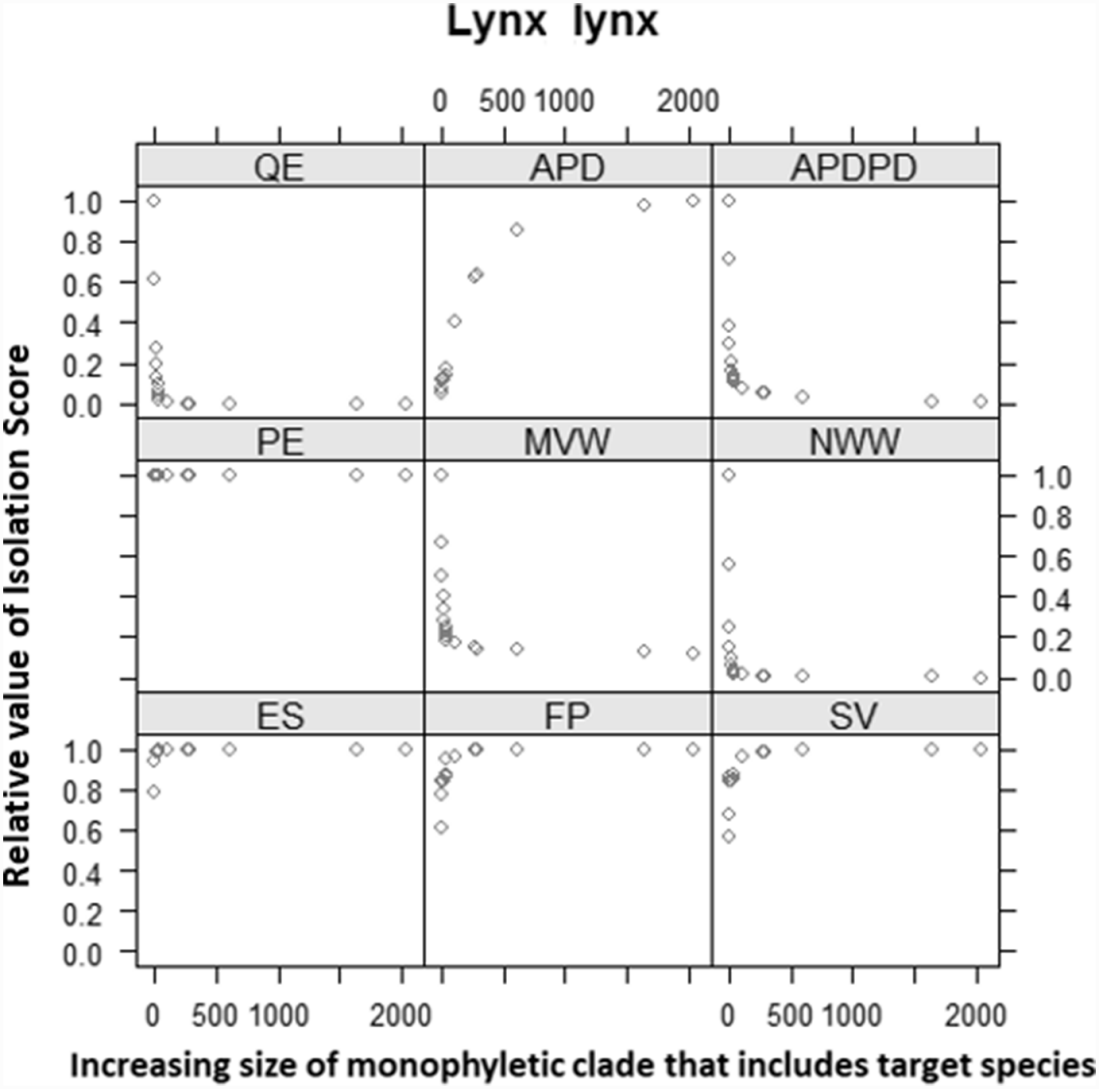
Eight different metrics of evolutionary isolation for a randomly chosen species (*Lynx lynx*) calculated on increasingly large complete clades that contain this target species. One metric APD appears twice, once in its original format and once as “APDPD,” where it is divided by the total PD of clade. All evolutionary isolation scores were standardised by dividing by the maximum score for that specific isolation metric. Metric acronyms are described in Table 1.

All evolutionary isolation metrics, save the pendant edge length (PE) are defined by a root and so are relative to some particular clade. This suggests that metrics correlated with PE may asymptote with increasing clade size while those that measure internal phylogenetic relationships may not. Indeed, when plotted (Fig. 5), for any particular tip, all metrics save APD surprisingly reached an apparent asymptote after a certain clade size, and standardizing the APD score by the total size of the tree (APD/PD) [31] brings this metric in line with the other measures (Fig. 5).

This effect of tree size on the absolute value of a metric score has important implications. To illustrate, if we estimate the slope for the upper asymptotic portion (300>×<3000) for each curve (as per Fig. 5) using linear models (*lm* function in R package *stats*) [25], we find that for mammal species average slope estimates range from β = −0.000124 for QE to β = 0.012168 for ES, and for amphibians average slope estimates ranged from β = −0.000006 for NWW to β = 0.008197 for ES. These estimates mean that even when using the measure with the steepest average slope (ES) a species with an evolutionary isolation score of 10 million years would only increase by approximately 0.1 million years to 10.1 million years if the ES score for that species was calculated on a full species level phylogeny that rooted one hundred nodes deeper into the tree of life.

Therefore, as a rule, if the clade on which an evolutionary isolation score for a particular target species is measured contains more than 250–300 species (or perhaps more precisely the species is more than 10–12 nodes from the root) then the resulting score is effectively absolute, for nearly all the metrics examined here (and including APD if it is weighted by total tree length). In this context evolutionary isolation scores (on almost any metric) for disparate large groups (birds vs. mammals vs. amphibians, for instance) can be compared directly for conservation ranking purposes, despite being calculated separately.

### Unifying Phylogenetic and Functional Species-level and Assemblage-level metrics of Biodiversity

Comparing functional and species based metrics on phylogenies reveals interesting parallels. In this work, we showed that the two dimensions found in phylogenetic isolation metrics (uniqueness and originality) match up well to the two different concepts in functional ecology: the ‘uniqueness’ and ‘originality’ of a species in functional space [10]. Furthermore, comparing community and species based metrics on phylogenies reveals more interesting parallels. The APD metric averaged across a community corresponds to the classical assemblage based mean pairwise distance (MPD). While towards the other end, ED, FP sums up to PD at the assemblage level. That said, more work is needed to understand the links between functional, assemblage and species level measure of biodiversity and to unify them in a common framework.

### Future work and conclusions

We highlight three potential avenues for future research. First, while it is clear that node-based metrics assign many species similar scores (Table 2), it is not clear whether subtle differences in isolation offered by other metrics are warranted. Given that trees are constantly being revised [32] ranks based on measures that differentiate all species (ED, QE) may be more sensitive to phylogenetic revision than metrics that give many species similar scores (NWU, MVW). Second, we have only compared the metrics on ultrametric trees, and their behavior on additive trees and networks has yet to be fully explored. For microbial organisms that readily exchange genetic information, for population-level data, or for ecological or morphological distances, networks are often more appropriate ways to represent relationships, and it may be that some metrics are more informative for these sorts of graphs. Finally, in this study, we focused on metrics based only on the phylogenetic position of species in the EDGE framework, ignoring other attributes such as the abundance or spatial extent of a species. Compound species-based metrics that incorporate such information (BED) [33], EDR [34]) have emerged or can be envisioned (e.g. a species-specific version of Rosauer’s phylogenetic endemism score PE) [35], and more work is required to understand the performance and sensitivity of these compound metrics.

Overall, it is important how evolutionary isolation is defined for conservation planners: for a given tree, the top 100 EDGE list could well be 50% (and up to 85%) different depending on the metric chosen. If the aim is to capture the phylogenetic information contained in a tree [36], or to increase the redundancy of already protected branches, or protect as much unique phylogenetic history as possible, then this aim needs to be made explicit and the methods by which the evolutionary isolation score is calculated matched with this aim. Without such clarity, we may be attempting, with very limited resources, to conserve species that do not represent what we want to conserve.
